# Oral Administration of [^18^F]MC225 for Quantification of P-glycoprotein Function: A Feasibility Study

**DOI:** 10.1007/s11307-024-01975-1

**Published:** 2025-01-14

**Authors:** Giordana Salvi de Souza, Cristiane R. G. Furini, Jürgen W. A. Sijbesma, Maria Kominia, Janine Doorduin, Bruno Lima Giacobbo, Adriaan A. Lammertsma, Charalampos Tsoumpas, Gert Luurtsema

**Affiliations:** 1 Department of Nuclear Medicine and Molecular Imaging, University Medical Center Groningen, University of Groningen, Groningen, The Netherlands; 2 School of Medicine, PUCRS, Porto Alegre, Brazil; 3 Laboratory of Cognition and Memory Neurobiology, Brain Institute, PUCRS, Porto Alegre, Brazil

**Keywords:** Biodistribution, Gastrointestinal tract, P-glycoprotein, Positron emission tomography

## Abstract

**Purpose:**

This preclinical study explored the feasibility of assessing P-glycoprotein (P-gp) function in both brain and gastrointestinal (GI) tract of rats using positron emission tomography (PET) following oral administration of [^18^F]MC225. Different oral administration protocols were evaluated, and radioactivity uptake was compared with uptake following intravenous administration.

**Procedures:**

Twelve male Wistar rats were divided into four groups and subjected to intravenous or oral [^18^F]MC225 administration protocols: G_1_ (intravenous route), G_2_ (oral administration without fasting), G_3_ (oral administration with fasting), and G_4_ (oral administration with fasting following administration of the P-gp inhibitor tariquidar). Dynamic brain imaging, late abdominal imaging, *ex vivo* biodistribution, and metabolite analysis were conducted to assess tracer distribution.

**Results:**

In the brain, oral administration yielded lower values compared with intravenous administration, resulting in a reduction in the tissue-to-plasma ratio by approximately 51% for the cortex and 45% for the midbrain and cerebellum. Fasting improved radioactivity uptake, aiding brain visualization. Unexpectedly, administration of the P-gp inhibitor tariquidar did not increase brain concentration, suggesting a signal that was dominated by non-specific uptake, possibly due to instability of [^18^F]MC225 in the GI tract. Metabolite analysis in G_4_ indicated a significant presence of polar metabolites.

**Conclusions:**

Oral administration of [^18^F]MC225 faces challenges and, at this stage, cannot be used to quantify P-gp function. Further research to assess tracer stability and metabolism in the stomach and intestine will be essential for advancing the feasibility of oral tracer administration.

**Supplementary Information:**

The online version contains supplementary material available at 10.1007/s11307-024-01975-1.

## Introduction

P-glycoprotein (P-gp) is a member of the adenosine triphosphate (ATP)-binding-cassette (ABC) transporter family, which acts as a membrane efflux pump [1]. It is primarily expressed in the liver, kidneys, gastrointestinal (GI) tract, pancreas, and the blood-brain barrier (BBB) [2]. P-gp expression in these sites impacts absorption, distribution, metabolism, and elimination of various substrates, including antibiotics, immunosuppressive agents, anticancer drugs, and more [3, 4]. In the GI tract, P-gp-mediated efflux across the apical membrane of enterocytes affects the rate and concentration of drugs diffusing across the basolateral membrane and entering general circulation from the intestine [5, 6]. Meanwhile, in the brain, P-gp acts as a gatekeeper, dynamically regulating the infiltration of substrates into the central nervous system (CNS), posing a critical challenge to the effectiveness of pharmacotherapies for neurological conditions [7].

The normal procedure for administering PET tracers is via intravenous (i.v.) injection. Although this procedure is the gold standard in both clinical practice and research studies, it limits the understanding of transport mechanisms from the intestinal epithelium into the GI tract lumen. Hence, oral administration of tracers has been proposed to investigate transport across various biological barriers, providing additional insights into the effects of transporters on drug tissue kinetics and delivery [8, 9]. However, challenges such as limited intestinal absorption, potential delays in organ uptake, and effects of food and acidity on tracer absorption must be addressed to ensure the reliability and reproducibility of tissue quantification obtained after oral administration [10, 11].

One method to quantify P-gp function involves the use of a radiolabeled P-gp substrate. It has been demonstrated that [^18^F]MC225, a specific and selective tracer of P-gp function, enables visualization of distribution and quantification of transport throughout the body using positron emission tomography (PET) [12]. While current clinical studies shed light on P-gp distribution in the brain [13–15], understanding how this distribution changes across the entire body and its relation to the GI tract poses an ongoing research challenge. Moreover, P-gp plays a dynamic role in determining the bioavailability of orally administered drugs [16, 17]. Furthermore, to understand why many drugs fail to reach the brain, it is crucial to determine whether the primary factor is their absorption through the intestinal epithelium or permeability of the BBB [6].

The aim of this preclinical pilot study was to assess whether oral administration of [^18^F]MC225 is a feasible option for PET imaging of both brain and GI tract. To this end, various oral administration protocols were evaluated, comparing uptake in brain and GI tract with that following intravenous administration.

## Methods

### [18F]MC225 Radiosynthesis

[^18^F]MC225 was manufactured at the Department of Nuclear Medicine and Molecular Imaging of the University Medical Center Groningen (EU-GMP production license: 108964 F). The molar activity was higher than 25,000 GBq/mmol and the radiochemical purity was higher than 98% in all cases. Production and quality control procedures of [^18^F]MC225 were performed as previously described [18, 19].

## Animals

This study was performed in accordance with the Animal Welfare Act of the European Communities Council Directive. The protocol was approved by the National Committee on Animal Experiments of the Netherlands and the Institutional Animal Care and Use Committee of the University of Groningen (CCD license: AVD105020198648, IvD protocol: 198648-01-005). All applicable institutional and/or national guidelines for the care and use of animals were followed.

Twelve adults male Wistar rats (Crl: WI(WU)) were obtained from Charles River Laboratories (Sulzfeld, Germany). Only male rats were selected to minimize the impact of the estrogen cycle on P-gp function at the BBB, as sex differences in the distribution of P-gp have been reported in animals [20].

Animals were acclimatized at the Central Animal Facility of the University Medical Center Groningen for at least 7 days before starting experiments. They were group housed in a temperature and humidity-controlled room with unlimited access to food and water.

## Experimental Design

In this pilot study, four groups of animals were used. The first group was the control group with a standard intravenous tracer injection. The other three groups received an oral administration of [^18^F]MC225, each group with a different protocol.

### Control Group: Intravenous Administration Group

The intravenous group, G_1_ (*n* = 3, body weight of 309 ± 8 g), was first anaesthetized with a mixture of 0.02 mL/g of ketamine and dexmedetomidine. This anesthetic combination was used because it has been shown not to alter intestinal motility compared with isoflurane, propofol, and pentobarbital [21, 22]. The animals were intravenously injected with 28.0 ± 8.0 MBq in a tail vein, while positioned in the PET scanner, using an infusion pump at a rate of 1 mL/min with a total administered volume of 1 mL.

### Oral Administration Groups

The oral administration methodology was based on a previous scoping review [8], which guided the selection of acquisition protocols to optimize tracer administration. Previous studies indicated that oral routes typically require approximately 40–70% of the radioactivity used for i.v. injections to reduce scattering and prevent high tracer concentrations in localized areas (e.g., stomach) [9, 10, 23, 24]. The gavage technique was selected for oral administration. This method delivers the tracer near the gastro-esophageal sphincter, allowing gravity to facilitate its entry into the stomach [25].

Tracer production for oral administration followed the same method as for i.v. administration [18, 19]. [^18^F]MC225, diluted in 1 mL saline, was administrated using disposable needles. For G_2_, the gavage needle used was Cadence Science 9918 (18G), from Fisher Scientific (Landsmeer, the Netherlands). However, close inspection indicated that changing the needle type could facilitate tracer administration more effectively, prompting a switch of needles. For one animal in G_3_ and the entire G_4_, the needles were substituted with FTP-15–78-SAM from Instech Laboratories (Plymouth, USA). Animals were awake during administration to monitor whether there were any complications. Following oral administration, 0.5 mL of saline was administered to flush the esophagus, ensuring that the tracer reached the stomach.

In the second group, G_2_ (*n* = 3 body weight 324 ± 4 g), the tracer was administered orally via gavage with an activity of 8.8 ± 1.7 MBq. Subsequently, animals were anesthetized using the same combination of anesthetics as for G_1_.

The third group, G_3_ (*n* = 3, body weight 302 ± 11 g), received the tracer orally via gavage as well, but with a higher activity of 17.0 ± 0.7 MBq. In addition, these animals underwent a fasting period of 12–14 h prior to the scan. The protocol modifications between G_3_ and G_2_ were introduced to assess whether higher radioactivity uptake, better image quality and more accurate quantification could be obtained.

To estimate whether oral administration could also detect any changes in P-gp function, the P-gp inhibitor tariquidar was administered in the fourth group. Previous studies using [^18^F]MC225 have indicated that doses higher than 3 mg/kg of tariquidar could significantly impact brain P-gp uptake in rats [15, 19]. To avoid the need for anesthesia, which would be needed for i.v. administration, tariquidar was administered intraperitoneally (i.p.). Previous studies have shown that the effects of tariquidar in rats remain constant and comparable to those after i.v. injection [26].

Tariquidar was prepared in a vehicle solution of 5% dimethyl sulfoxide (DMSO), 10% TWEEN 20, 25% polyethylene glycol 400 (PEG400), and 65% H_2_O, following the same protocol as for i.v. administration [15]. A dose of 10 mg/kg, selected as an intermediate value based on previous studies, was administered [15]. Group G_4_ (*n* = 3, body weight 286 ± 30 g) received a dose of 10.1 ± 1.6 mg/kg of tariquidar, with an average volume of 0.86 ± 0.21 mL (Bio-Techne Ltd., Abingdon, UK), 1 h before tracer administration. This timing was chosen to ensure that tariquidar’s peak concentration, which occurs 2 h after i.p. injection, coincided with tracer acquisition [26].

The tracer, with an activity of 16.6 ± 3.2 MBq, was administered via oral gavage following a 12–14 h fasting period prior to the scan. Table 1 summarizes the groups, body weights, administered activities and routes, and time between tracer administration and start of scan.

**Table 1:** Description of experimental groups and administration protocols. Results expressed in mean ± SD, and [range]

Group	Body weight (g)	Age (weeks)	Activity (MBq)	Activity (MBq/g)	Administration Route	Delay (min)^1^	Additional information
G_1_	309.1 ± 10.1 [18.7]	9.3 ± 0.5 [1]	28.0 ± 9.8 [17.9]	31.0 ± 9.4 [18.0]	Intravenous	0	-
G_2_	323.6 ± 4.9 [9.6]	10 ± 0.0 [0]	8.8 ± 2.1 [3.9]	15.6 ± 4.1 [8.1]	Oral	6.3 ± 0.5	-
G_3_	295.2 ± 15.14 [29.4 ]	10.3 ± 0.6 [1.0]	18.7 ± 2.9 [5.5]	29.3 ± 5.3 [11.2]	Oral	12.5 ± 0.9	12–14 h fasting
G_4_	285.6 ± 30.4 [73.8]	9.7 ± 1.2 [3.0]	16.6 ± 3.9 [7.1]	15.9 ± 2.8 [5.2]	Oral	13 ± 7.1	12–14 h fasting; tariquidar (i.p) 1 h before tracer administration

^1^Time between tracer administration and start of scan

## PET Acquisitions

Dynamic brain scans (microPET Focus 220, Siemens Medical Solutions, Malvern, USA) were started simultaneously with tracer injection (G_1_) or immediately after animals were anesthetized following oral tracer administration (G_2_-G_4_) (Table 1). In G_1_ and G_2_, a 90 min dynamic scan was followed by an 8 min transmission scan using a ^57^Co point source for attenuation and scatter correction. In G_3_ and G_4_, a 240 min dynamic scan was performed, followed by a similar transmission scan. In addition, in all groups, a 15 min abdominal PET scan followed by a transmission scan was performed immediately after the brain scan.

To ensure physiological stability, body temperature was maintained using a heating pad and electronic temperature controller for precise regulation. The pad was placed underneath the animal, and the controller monitored rectal temperature via a probe, ensuring it was within a physiological range of 37 °C. Blood oxygenation levels were tracked with pulse oximeter on the paw, and pulse rate was recorded. Throughout all scans, oxygenation, temperature, and pulse rate were continuously monitored to ensure animal well-being and data quality.

## PET Reconstructions

Brain PET scans of G_1_ and G_2_ were histogrammed into 24 frames: 6 × 10, 4 × 30, 2 × 60, 1 × 120, 1 × 180, 4 × 300 and 6 × 600 s. For G_3_ and G_4_, brain PET scans were histogrammed into 20 frames of 720 s. This frame time was suitable as the uptake in these groups was observed to be low and constant throughout the scan duration. Abdomen PET data were histogrammed into a single frame lasting 15 min. Image reconstruction was performed using the Fourier rebinning, high-resolution, two-dimensional ordered-subset expectation maximization (2D OSEM) algorithm with 4 iterations and 16 subsets, after being normalized and corrected for attenuation, scatter, dead time, random coincidences, and radioactivity decay. Reconstructed images were sampled over a grid of 256 × 256 × 95 voxels, with a zoom of 1.5 and a voxel size of 0.633 × 0.633 × 0.796 mm^3^.

## *Ex Vivo* Biodistribution

After scanning, animals were terminated by extirpation of the heart under anesthesia. Whole blood, plasma and tissue samples from various brain regions (cortex, midbrain, cerebellum), GI organs (stomach, duodenum, jejunum, ileum, cecum, colon) [27], and other organs and tissues (heart, lungs, pancreas, spleen, kidneys, adrenals, liver, muscle, femur) were collected for *ex vivo* analysis. GI contents were removed prior to tissue measurements. These samples were weighed, and radioactivity concentrations were measured using a gamma counter (2480 WIZARD 2, PerkinElmer, Waltham, USA), expressed as kBq/g of tissue. The tissue-to-plasma ratio was calculated to assess the concentration within tissue, relative to plasma, considering the diffusion of the tracer from the GI tract into the bloodstream.

### Metabolite Analysis

For one animal, from G_4_, the plasma parent fraction was determined following the procedure outlined by Savolainen et al. (2017) [19]. Plasma was precipitated by adding two volumes of acetonitrile, followed by centrifugation for 5 min at 6000 rpm. Supernatant samples were then spotted on a thin layer chromatography (TLC, F-254 silica gel plates, Sigma–Aldrich) plate, which subsequently was eluted with 10% methanol in ethyl acetate. Radioactivity on the plates was detected using phosphor storage imaging using a Cyclone^®^ system (PerkinElmer Life and Analytical Science, Waltham, USA). The fractions of intact tracer and radioactive metabolites were calculated by region of interest (ROI) analysis using ImageQuant TL software (PerkinElmer, Waltham, MA, USA).

## Data Analysis

PET images were co-registered with an [^18^F]MC225-specific brain template for Wistar rats using rigid matching [28] and then analyzed using PMOD v4.1 (PMOD Technologies, Zurich, Switzerland). Predefined brain regions were selected as volumes of interest (VOI), and tissue radioactivity (kBq/mL) was determined to generate tissue time-activity curves (TACs).

Standardized uptake value (SUV) images were generated using the following formula:

$$\:SUV=\frac{\text{T}\text{i}\text{s}\text{s}\text{u}\text{e}\:\text{a}\text{c}\text{t}\text{i}\text{v}\text{i}\text{t}\text{y}\:\text{c}\text{o}\text{n}\text{c}\text{e}\text{n}\text{t}\text{r}\text{a}\text{t}\text{i}\text{o}\text{n}\:(\text{k}\text{B}\text{q}/\text{m}\text{L})}{\frac{\text{I}\text{n}\text{j}\text{e}\text{c}\text{t}\text{e}\text{d}\:\text{d}\text{o}\text{s}\text{e}\:\left(\text{M}\text{B}\text{q}\right)}{\text{B}\text{o}\text{d}\text{y}\:\text{w}\text{e}\text{i}\text{g}\text{h}\text{t}\:\left(\text{k}\text{g}\right)}}$$

### *In Vitro* Experiment

An *in vitro* experiment was conducted to evaluate the stability of [^18^F]MC225 in the stomach, mimicking low pH and enzymatic conditions as described previously [29]. The pH was maintained at 1.7 in both experiments. Enzymatic conditions were assessed using pepsin, a stomach enzyme that serves to digest proteins that are present in ingested food [30].

The gastric fluid without the enzyme was prepared by dissolving 20 mg of NaCl (sodium chloride) in 70 µL of (12 N)HCl (hydrochloric acid) and 10 mL of water. Simulated gastric fluid was prepared by dissolving 20 mg of NaCl and 32 mg of pepsin from porcine gastric mucosa (≥ 2500 units/mg) in 70 µL of (12 N)HCl and 10 mL of water.

1 mL of each solution was added to a microtube and heated to 37 °C. 50 µL of [^18^F]MC225 was added, and the resulting mixture was stirred at 300 rpm and 37 °C for 2 h. Samples were taken every 30 min and analyzed using TLC according to the same procedure as for the metabolites.

### Statistics

Descriptive data are presented as mean ± standard deviation (SD) unless otherwise specified.

## Results

### Analysis of PET Data

**Fig. 1:**
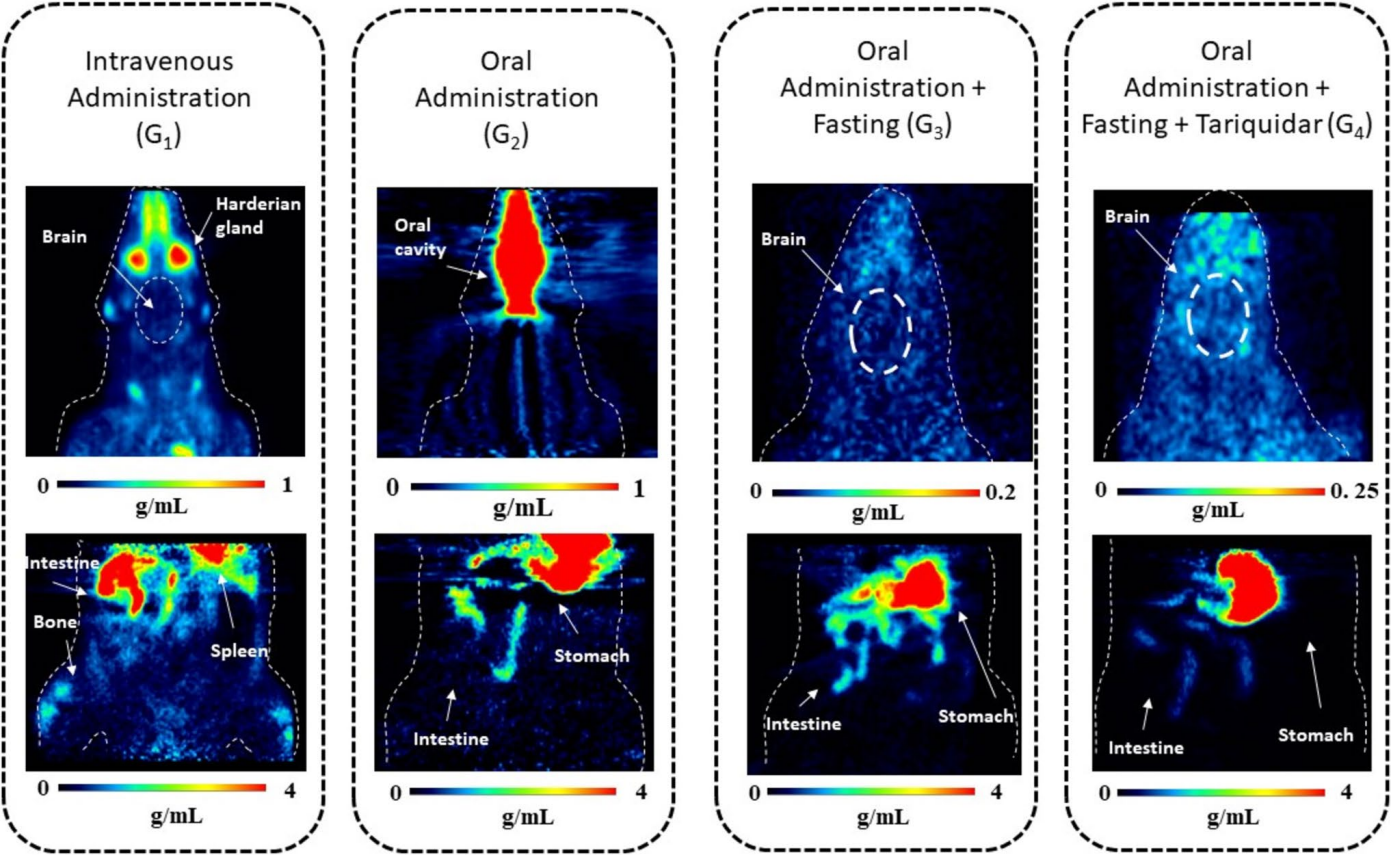
Coronal slices through brain images for each group are presented in standardized uptake values (SUV), in g/mL. Static images were acquired from 0 to 90 min for G_1_ and G_2_, and from 0 to 96 min for G_3_ and G_4_. In addition, 15 min static [^18^F]MC225 PET images of the abdomen are depicted. Abdominal scans are not directly comparable between groups due to differences in acquisition time: for G_1_ and G_2_, scans were acquired 98 min after tracer administration, while for G_3_ and G_4_, scans were acquired 248 min after tracer administration

Figure 1 illustrates both brain and abdominal images for each group in this study. For comparison purposes, attempts were made to establish static brain images with the same time interval between i.v. and oral administration groups. It is important to note that selection of each animal in this figure was based on visual clarity of the image, rather than on radioactivity uptake in the brain. In addition, radioactivity uptake was observed in abdominal organs even after i.v. administration. The supplementary material shows all brain and abdominal PET acquisitions of all animals in three planes: axial, coronal, and sagittal (Supplementary Figs. 1–5).

**Fig. 2:**
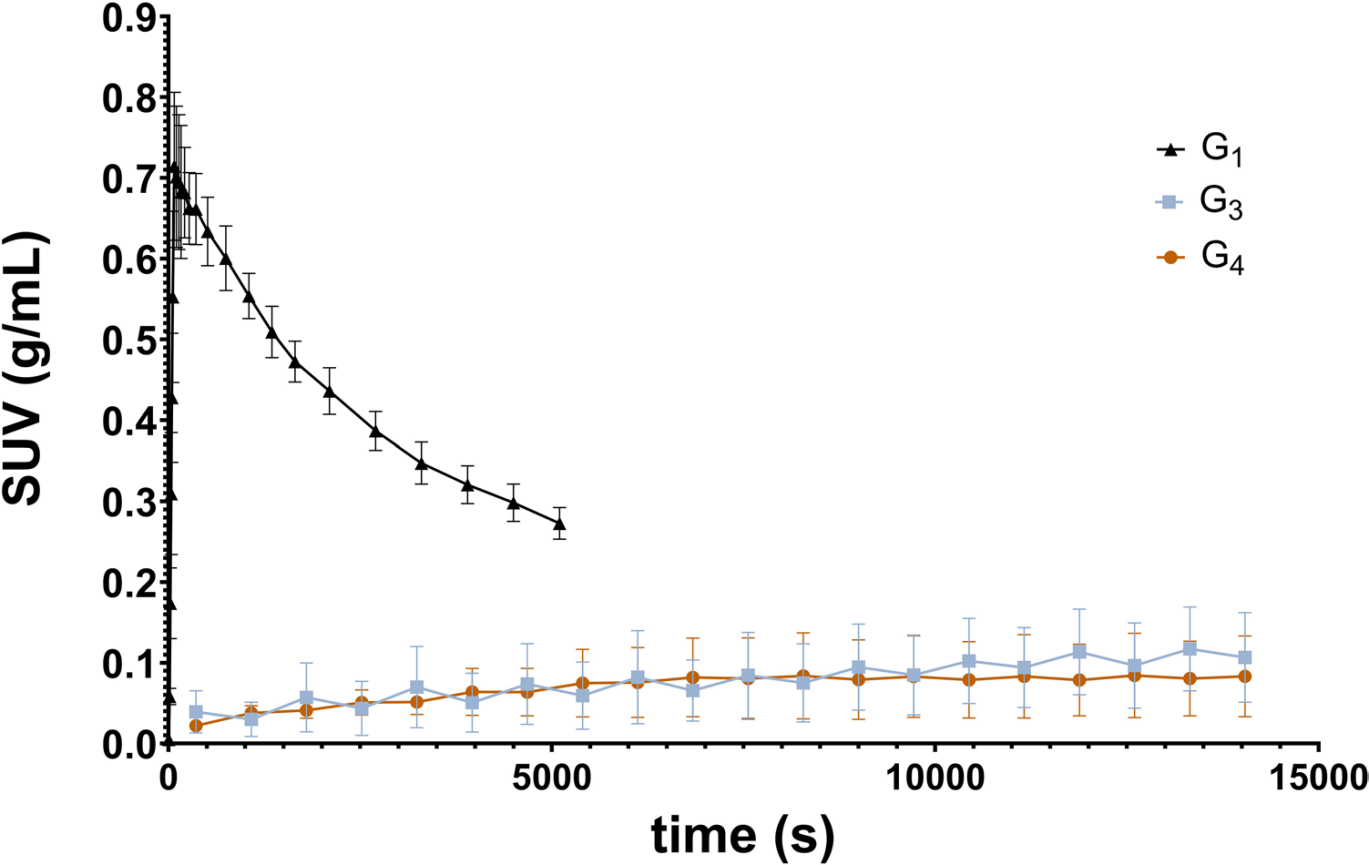
TACs for the whole brain region. G_1_: i.v. injection (90 min of PET acquisition); G_3_: oral administration with 12–14 h fasting (240 min of PET acquisition); G_4_: oral administration with 12–14 h fasting and tariquidar i.p. injection (240 min of PET acquisition)

Visualizing the brain in the oral administration group G_2_ was not possible, therefore TACs were not extracted. The esophagus exhibited high radioactivity uptake in the PET image, likely due to the gavage technique, potentially resulting in some spillover effect. However, this does not imply a lack of brain radioactivity uptake; rather, the high tracer concentration in the esophagus obscured the delineation of brain regions. To address these challenges in the subsequent group (G_3_), several measures were implemented. For one animal, a longer needle for oral gavage was introduced, aiming to deliver the tracer closer to the stomach, thus preventing its deposition in the esophagus. In addition, for all animals, fasting was implemented to reduce stomach content, thereby enhancing tracer distribution, especially in the brain. These adjustments facilitated the registration of the PET image template and enabled the subsequent extraction of TACs for each brain region evaluated. In Fig. 2, the average TACs of the whole brain for groups G_1_, G_3_, and G_4_ are presented.

Low but constant radioactivity uptake in the brain was observed in G_3_ and G_4_. Administration of tariquidar, a P-gp inhibitor, did not result in higher SUV values in the brain (G_4_vs. G_3_).

### *Ex Vivo* Biodistribution

The time between tracer injection and counting of radioactivity was recorded for each group. For G_1_, the time was 174 ± 18 min; for G_2_, it was 178 ± 4 min, and for G_3_ and G_4_, the times were 333 ± 10 and 333 ± 14 min, respectively.

**Fig. 3:**
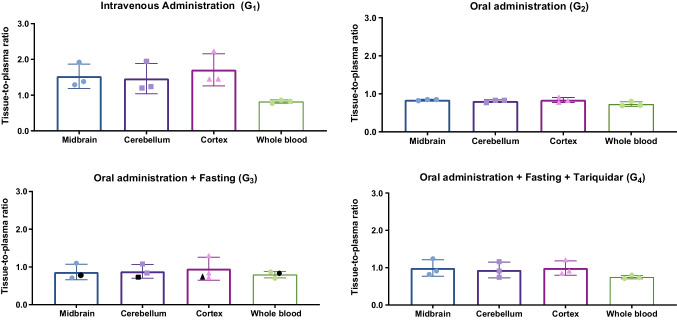
*Ex vivo *results for brain and whole blood (mean ± SD). Results are expressed in terms of tissue-to-plasma ratio. The animal in G_3_ plotted in black received the oral gavage with a longer needle. For G_1_ the time between tracer administration and biodistribution was 174 ± 18 min, for G_2_ it was 178 ± 4 min, and for G_3_ and G_4_ the time was 333 ± 10 min and 333 ± 14 min, respectively

**Fig. 4:**
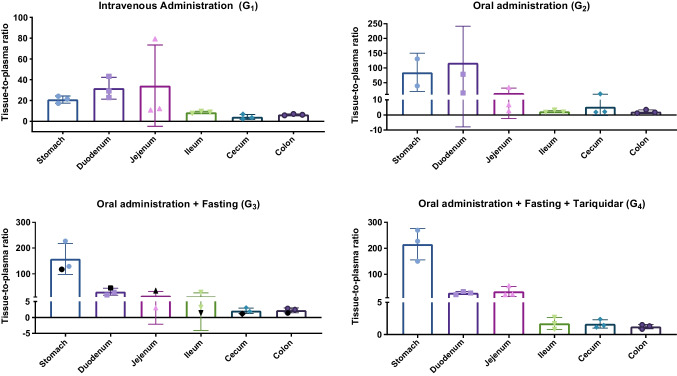
*Ex vivo* results for GI tract (mean ± SD). Results are expressed in terms of tissue-to-plasma ratio. The animal in G_3_ plotted in black received the oral gavage with a longer needle. For G_1_ the time between tracer administration and biodistribution was 174 ± 18 min, for G_2_ it was 178 ± 4 min, and for G_3_ and G_4_ the time was 333 ± 10 min and 333 ± 14 min, respectively

**Fig. 5:**
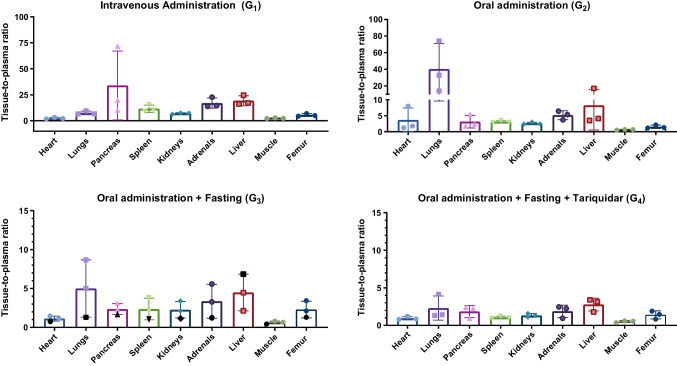
*Ex vivo* results for other organs and tissues (mean ± SD). Results are expressed in terms of tissue-to-plasma ratio. The animal in G_3_ plotted in black received the oral gavage with a longer needle. For G_1_ the time between tracer administration and biodistribution was 174 ± 18 min, for G_2_ it was 178 ± 4 min, and for G_3_ and G_4_ the time was 333 ± 10 and 333 ± 14 min, respectively

 Overall, oral administration resulted in lower brain tracer concentrations compared with i.v. injection, by approximately 51% for the cortex and 45% for the midbrain and cerebellum. Figures 3, 4 and 5 present *ex vivo* results for brain and whole blood (Fig. 3), the GI tract (Fig. 4), and other organs and tissues (Fig. 5), adjusting for changes in delivery (plasma concentration). The supplementary material shows the results in terms of kBq/g and tissue-to-plasma ratios (Tables 1 and 2).

In the tariquidar-treated group (G_4_), there was an increase of 4% in the cortex, 13% in midbrain, and 6% in cerebellum compared with G_3_. It should be noted, however, that despite the increase in tissue-to-plasma ratios in G_4_ compared with G_3_, actual brain tracer concentrations decreased, as plasma levels were much lower in G_4_ than in G_3_. For the other organs and tissues evaluated, there were no obvious differences between groups G_3_ and G_4_.

### Metabolite Analysis

Results for the G_4_ prompted an investigation into plasma metabolites, aiming to elucidate the reasons behind the absence of increased brain radioactivity uptake observed after the use of tariquidar. This revealed that only 4.1% of the total radioactivity in plasma was related to the parent tracer, with a dominant presence of labelled polar metabolites (91%). Supplementary Fig. 6 shows TLC plates with the respective analyses.

### *In Vitro* Experiment

[^18^F]MC225 showed stability under simulated gastric conditions for up to 2 h, with no significant decomposition observed in the acidic environment. The amount of intact tracer at *t* = 0 min corresponded with the radiochemical purity, which was higher than 95%. In the gastric fluid without pepsin, 86% of the tracer remained intact after 30 min, with no significant change (remaining 88%) after 120 min. In the solution containing pepsin, the tracer stability was slightly higher, with 92% intact after 30 min, and a similar pattern of stability observed over time, ending with 92% intact after 120 min.

## Discussion

This preclinical PET imaging study assessed the feasibility of orally administering the P-gp substrate [^18^F]MC225 and quantifying it for both the brain and GI tract. P-gp, as a crucial regulator of drug concentrations in the blood, brain, and other tissues, plays a pivotal role in drug transport across the basolateral membrane. Understanding P-gp function in the intestine is particularly relevant for understanding variations in drug treatment, especially in pharmacoresistant patients [7].

As anticipated, oral administration yielded lower tracer concentrations in the brain compared with i.v. administration (G_1_vs. G_2_) [8]. Methodological changes, such as varying the amount of administered activity, using longer needles, animal fasting and modifying the acquisition duration, were explored to optimize tracer quantification. For the brain, these modifications resulted in higher uptake and better visualization (G_3_vs. G_2_), and this was confirmed by the *ex vivo* results, even with a difference of 2.5 h (Fig. 3).

Analysis of the GI tract images was not conducted due to challenges of distinguishing the tracer signal within the GI lumen from that related to transport within the bloodstream. Notably, the change of needle size had a significant impact on the tracer distribution. Deposition of the tracer in the esophagus is related to the concept of oral administration by gavage. With this technique, the tracer is delivered near the gastro-esophageal sphincter rather than directly into the stomach, as it is done with gastrostomy. Although the administration of the tracer was slow, esophageal reflux occurred [31] and the lipophilic and sticky nature of [^18^F]MC225 may have contributed to its adherence to the tissue. While switching to a longer needle may not yield benefits for non-radioactive compounds and could increase the risk of perforation, it proved advantageous in imaging studies involving radioactive compounds.

Contrary to expectations, the group receiving the P-gp inhibitor tariquidar (G_4_) did not exhibit a higher brain concentration than those without (G_3_). Higher tracer concentration was expected as inhibiting the P-gp transporter typically leads to increased tracer diffusion into cells, particularly in the brain and plasma, as demonstrated in previous studies [15, 19]. This unexpected finding suggests that the observed signal is dominated by non-specific uptake. Indeed, analysis of metabolites in plasma showed a high fraction of small labeled polar metabolites that reach the whole blood compartment via diffusion. Five hours after administration, only 4.1% of the total radioactivity in plasma represented intact tracer, supporting the hypothesis that the signal in the brain was dominated by radiolabeled metabolites rather than parent [^18^F]MC225. However, it is not possible to draw definitive conclusions from this observation, as it was derived from one blood sample obtained from one animal five hours after tracer administration. Interestingly, radioactivity concentration in the bone (femur) was attributed to defluorination during [^18^F]MC225 metabolism as previously described [19]. For the oral administration route, the tissue-to-plasma ratio of bone was lower compared with the i.v. route, indicating that metabolism of the tracer differed between these two administration methods.

*In vitro* stability studies revealed that [^18^F]MC225 is stable under simulated gastric conditions, suggesting that the challenges associated with oral administration of [^18^F]MC225 are not due to instability in the stomach. This stability under gastric conditions indicates that the breakdown and formation of metabolites likely occur when the tracer reaches the intestine. One possible explanation is that enterocytes, which express P-gp and enzymes such as CYP3A4, play a significant role in drug metabolism within the GI tract [32]. P-gp and CYP3A4 are co-localized in enterocytes, share common substrates, and are co-inducible in response to some xenobiotics [32]. It is likely that [^18^F]MC225 enters the enterocyte via diffusion and is promptly metabolized before being expelled from the cell via the P-gp efflux pump. This metabolic process of uptake by diffusion and efflux by active transport occurs throughout the small intestine, which could partially explain the oscillation observed in the tissue TAC of G_3_. This effect is not seen when the P-gp inhibitor tariquidar is administered in conjunction with the radioligand in G_4_ (Fig. 2). Future studies are warranted to comprehensively investigate the uptake and metabolism of [^18^F]MC225 in the intestine to elucidate these dynamics further.

While the present results do not support the feasibility of oral administration of [^18^F]MC225, they underscore the significant challenges associated with this method, particularly concerning metabolizing enzymes in the GIT. A repeated G_2_ study could potentially improve the groups’ comparisons regarding the feasibility of administering tracers via this route. However, the principles of the 3Rs (Replacement, Reduction and Refinement) in animal research did not justify additional experiments. Although the PET images from this study were not of satisfactory quality because of high radioactivity concentration in the esophagus, conclusions were drawn from the corresponding *ex vivo* biodistribution results and the comparison between G_3_ and G_4_. Continuing the study would subject the animals to unnecessary procedures and discomfort without yielding directly meaningful or beneficial data.

Prior to further PET scans, rigorous *in vitro* studies are essential to assess tracer stability and metabolism in both the stomach and intestine. These studies are crucial for advancing the feasibility and reliability of oral tracer administration. Moreover, utilizing the already available 3D organoids system of small intestine could provide valuable insights into the impact of P-gp function on the absorption and metabolization of [^18^F]MC225 within the intestine, particularly concerning the effects of P-gp dysfunction [33, 34].

## Conclusions

Oral administration of [^18^F]MC225 resulted in an unexpected high level of radiolabeled metabolites together with the lack of a response to P-gp inhibition, warrant furthering investigation into tracer metabolism. These findings underscore the importance of a better understanding of GI tract physiology and its impact on tracer absorption and breakdown to refine oral administration protocols and optimize tracer delivery to target tissues.

## Electronic Supplementary Material

Below is the link to the electronic supplementary material.

Supplementary Material 1

## Data Availability

All research data are available from the corresponding author upon request.

## References

[1] Kapoor K, Pant S, Tajkhorshid E (2021) Active participation of membrane lipids in inhibition of multidrug transporter P-glycoprotein. Chem Sci J 12:6293–6306. 10.1039/D0SC06288J 10.1039/d0sc06288j PMC8115088 34084427

[2] Liu Y, Hu M (2000) P-Glycoprotein and bioavailability-implication of polymorphism. Clin Chem Lab Med 38:877–881. 10.1515/CCLM.2000.127 11097343 10.1515/CCLM.2000.127

[3] Ahmed Juvale II, Abdul Hamid AA, Abd Halim KB, Che Has AT (2022) P-glycoprotein: new insights into structure, physiological function, regulation and alterations in disease. Heliyon 8:e09777. 10.1016/j.heliyon.2022.e09777 35789865 10.1016/j.heliyon.2022.e09777 PMC9249865

[4] Alfarouk KO, Stock C-M, Taylor S et al (2015) Resistance to cancer chemotherapy: failure in drug response from ADME to P-gp. Cancer Cell Int 15:71. 10.1186/s12935-015-0221-1 26180516 10.1186/s12935-015-0221-1 PMC4502609

[5] Murakami T, Bodor E, Bodor N (2020) Modulation of expression/function of intestinal P-glycoprotein under disease states. Expert Opin Drug Metab Toxicol 16:59–78. 10.1080/17425255.2020.1701653 31821048 10.1080/17425255.2020.1701653

[6] Hoosain FG, Choonara YE, Tomar LK et al (2015) Bypassing P-Glycoprotein drug efflux mechanisms: possible applications in Pharmacoresistant Schizophrenia Therapy. Biomed Res Int 2015:1–21. 10.1155/2015/484963 10.1155/2015/484963 PMC4600488 26491671

[7] Bartels L, de Klerk AL, Kortekaas O et al (2010) 11 C-verapamil to assess P-gp function in human brain during aging,Depression and neurodegenerative disease. Curr Top Med Chem 10:1775–1784. 10.2174/156802610792928059 20645917 10.2174/156802610792928059

[8] Salvi de Souza G, Mantovani DBA, Mossel P et al (2023) Oral administration of PET tracers: current status. J Control Release 357:591–605. 10.1016/j.jconrel.2023.04.008 37031742 10.1016/j.jconrel.2023.04.008

[9] Kataoka M, Takashima T, Shingaki T et al (2012) Dynamic analysis of GI absorption and hepatic distribution processes of Telmisartan in rats using Positron Emission Tomography. Pharm Res 29:2419–2431. 10.1007/s11095-012-0768-7 22618800 10.1007/s11095-012-0768-7

[10] Higashi T, Fisher SJ, Nakada K et al (2002) Is enteral administration of fluorine-18-fluorodeoxyglucose (F-18 FDG) a palatable alternative to IV injection? Nucl Med Biol 29:363–373. 10.1016/S0969-8051(01)00312-2 11929708 10.1016/s0969-8051(01)00312-2

[11] Shingaki T, Katayama Y, Nakaoka T et al (2016) Exploration of Antiemetics for osteoporosis Therapy-Induced nausea and Vomiting Using PET Molecular Imaging Analysis to gastrointestinal pharmacokinetics. Pharm Res 33:1235–1248. 10.1007/s11095-016-1868-6 26869173 10.1007/s11095-016-1868-6

[12] Mossel P, Garcia Varela L, Arif WM et al (2021) Evaluation of P-glycoprotein function at the blood–brain barrier using [18F]MC225-PET. Eur J Nucl Med Mol Imaging 48:4105. 10.1007/s00259-021-05419-8 34089347 10.1007/s00259-021-05419-8 PMC8484189

[13] Toyohara J, Sakata M, Ishibashi K et al (2021) First clinical assessment of [18F]MC225, a novel fluorine-18 labelled PET tracer for measuring functional P-glycoprotein at the blood–brain barrier. Ann Nucl Med 35:1240. 10.1007/s12149-021-01666-9 34368924 10.1007/s12149-021-01666-9

[14] Mossel P, Arif WM, De Souza GS et al (2023) Quantification of P-glycoprotein function at the human blood-brain barrier using [18F]MC225 and PET. Eur J Nucl Med Mol Imaging. 10.1007/s00259-023-06363-5 37552369 10.1007/s00259-023-06363-5 PMC10611838

[15] Garcia-Varela L, Mossel P, Aguiar P et al (2022) Dose-response assessment of cerebral P-glycoprotein inhibition *in vivo* with [18F]MC225 and PET. J Control Release 347:500–507. 10.1016/j.jconrel.2022.05.026 35588934 10.1016/j.jconrel.2022.05.026

[16] Shugarts S, Benet LZ (2009) The role of transporters in the pharmacokinetics of orally administered drugs. Pharm Res 26:2039–2054. 10.1007/s11095-009-9924-0 19568696 10.1007/s11095-009-9924-0 PMC2719753

[17] Gavhane YN, Yadav AV (2012) Loss of orally administered drugs in GI tract. Saudi Pharm J 20:331–344. 10.1016/j.jsps.2012.03.005 23960808 10.1016/j.jsps.2012.03.005 PMC3744959

[18] Savolainen H, Cantore M, Colabufo NA et al (2015) Synthesis and preclinical evaluation of three Novel Fluorine-18 labeled Radiopharmaceuticals for P-Glycoprotein PET imaging at the blood-brain barrier. Mol Pharm 12:2265. 10.1021/mp5008103 26043236 10.1021/mp5008103

[19] Savolainen H, Windhorst AD, Elsinga PH et al (2017) Evaluation of [^18^ F]MC225 as a PET radiotracer for measuring P-glycoprotein function at the blood–brain barrier in rats: kinetics, metabolism, and selectivity. J Cereb Blood Flow Metab 37:1286–1298. 10.1177/0271678X16654493 27354093 10.1177/0271678X16654493 PMC5453451

[20] Madla CM, Qin Y, Gavins FKH et al (2022) Sex differences in intestinal P-Glycoprotein expression in Wistar versus Sprague Dawley rats. Pharmaceutics 14:1030. 10.3390/pharmaceutics14051030 35631615 10.3390/pharmaceutics14051030 PMC9143158

[21] Schreiber D, Markus K, Kerstin L et al (2014) The mesenterially perfused rat small intestine: a versatile approach for pharmacological testings. Annals Anat 196. 10.1016/j.aanat.2014.02.008 10.1016/j.aanat.2014.02.008 24690290

[22] Albrecht M, Henke J, Tacke S et al (2014) Influence of repeated anaesthesia on physiological parameters in male Wistar rats: a telemetric study about isoflurane, ketamine-xylazine and a combination of medetomidine, midazolam and fentanyl. BMC Vet Res 10:310. 10.1186/s12917-014-0310-8 10.1186/s12917-014-0310-8 PMC4301083 25551200

[23] Ding Y, Gatley SJ, Thanos PK et al (2004) Brain kinetics of methylphenidate (ritalin) enantiomers after oral administration. Synapse 53:168–175. 10.1002/syn.20046 15236349 10.1002/syn.20046

[24] Yamashita S, Takashima T, Kataoka M et al (2011) PET imaging of the gastrointestinal absorption of orally administered drugs in conscious and anesthetized rats. J Nucl Med 52:249–256. 10.2967/jnumed.110.081539 21233189 10.2967/jnumed.110.081539

[25] Eichenbaum G, Damsch S, Looszova A et al (2011) Impact of gavage dosing procedure and gastric content on adverse respiratory effects and mortality in rat toxicity studies. J Appl Toxicol 31:342–354. 10.1002/jat.1592 21089156 10.1002/jat.1592

[26] Matzneller P, Kussmann M, Eberl S et al (2018) Pharmacokinetics of the P-gp inhibitor tariquidar in rats after intravenous, oral, and Intraperitoneal Administration. Eur J Drug Metab Pharmacokinet 43:599–606. 10.1007/s13318-018-0474-x 29616423 10.1007/s13318-018-0474-x PMC6133083

[27] DeSesso JM, Jacobson CF (2001) Anatomical and physiological parameters affecting gastrointestinal absorption in humans and rats. Food Chem Toxicol 39:209–228. 10.1016/S0278-6915(00)00136-8 11278053 10.1016/s0278-6915(00)00136-8

[28] García-Varela L, Vállez García D, Rodríguez-Pérez M et al (2020) Test–retest repeatability of [ ^18^ F]MC225-PET in rodents: a Tracer for imaging of P-gp function. ACS Chem Neurosci 11:648–658. 10.1021/acschemneuro.9b00682 31961646 10.1021/acschemneuro.9b00682 PMC7034080

[29] Pharmacopeia US (2006) The United States pharmacopeia: USP 29; the National formulary: NF 24. US Pharmacopeial Convention, Rockville,Maryland

[30] Rowbotham DJ, Kimpson PM, Thompson HM (2006) Gut motility and secretions. In: Hemmings HC, Hopkins PM (eds) Foundations of anesthesia, 2nd edn. Elsevier, pp 739–751. 10.1016/B978-0-323-03707-5.50067-X. https://www.sciencedirect.com/science/article/pii/B978032303707550067X

[31] Damsch S, Eichenbaum G, Tonelli A et al (2011) Gavage-related reflux in rats. Toxicol Pathol 39:348–360. 10.1177/0192623310388431 21422261 10.1177/0192623310388431

[32] Watkins P (1997) The barrier function of CYP3A4 and P-glycoprotein in the small bowel. Adv Drug Deliv Rev 27:161–170. 10.1016/S0169-409X(97)00041-0 10837556 10.1016/s0169-409x(97)00041-0

[33] Yoshida S, Miwa H, Kawachi T et al (2020) Generation of intestinal organoids derived from human pluripotent stem cells for drug testing. Sci Rep 10:5989. 10.1038/s41598-020-63151-z 32249832 10.1038/s41598-020-63151-z PMC7136241

[34] Zhao J, Zeng Z, Sun J et al (2017) A novel model of P-Glycoprotein inhibitor screening using human small intestinal organoids. Basic Clin Pharmacol Toxicol 120:250–255. 10.1111/bcpt.12680 27657920 10.1111/bcpt.12680

