# Supplementary material for: Oral Administration of [18F]MC225 for Quantification of P-glycoprotein Function: A Feasibility Study
Source: Mol Imaging Biol. 2025 Jan 14;27(1):89–98. doi: 10.1007/s11307-024-01975-1 (PMC11805767; doi:10.1007/s11307-024-01975-1)
Supplement: Supplementary file 1 — Supplementary Material 1 [file 11307_2024_1975_MOESM1_ESM.pdf]

Electronic Supplementary Material

Oral Administration of [^{18}F]MC225 for Quantification of P-glycoprotein Function: A Feasibility Study

Giordana Salvi de Souza[1,2], Cristiane R. G. Furini[2,3], Jürgen W. A. Sijbesma[1], Maria Kominia[1], Janine Doorduin[1], Bruno Lima Giacobbo[1], Adriaan A. Lammertsma[1], Charalampos Tsoumpas[1], Gert Luurtsema[1]

[1] Department of Nuclear Medicine and Molecular Imaging, University Medical Center Groningen, University of Groningen, Groningen, The Netherlands

[2] School of Medicine, PUCRS, Porto Alegre, Brazil

[3] Laboratory of Cognition and Memory Neurobiology, Brain Institute, PUCRS, Porto Alegre, Brazil

Corresponding author:

Gert Luurtsema

Groningen, Groningen, the Netherlands

E-mail: g.luurtsema@umcg.nl

Phone: +31 50 361 3541

Running title: Oral Administration of [^{18}F]MC225

Manuscript Category: Original article

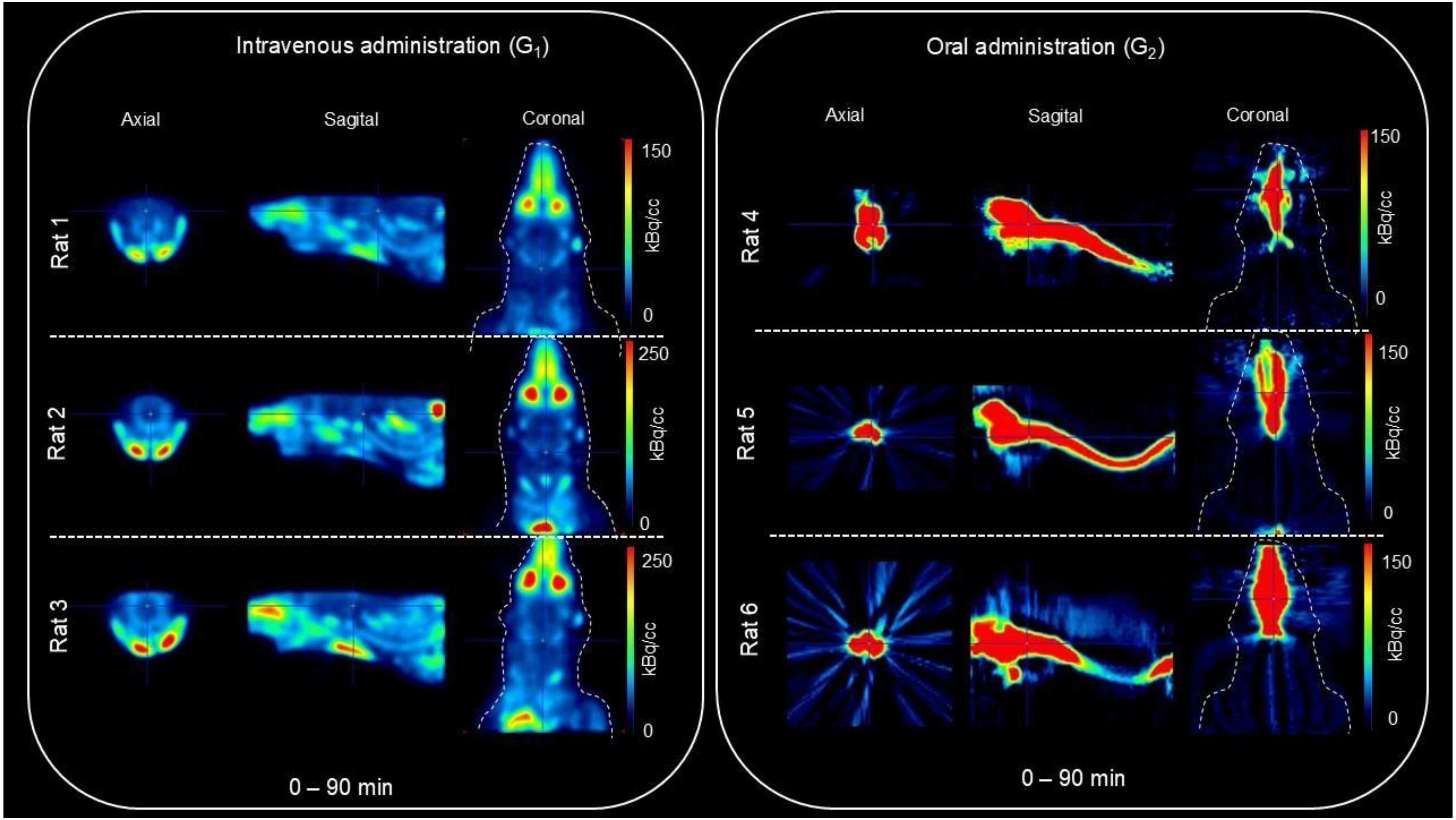

Supplementary Figure 1. Brain PET scan for group G_1 and G_2. G_1: intravenous injection; G_2: oral administration.

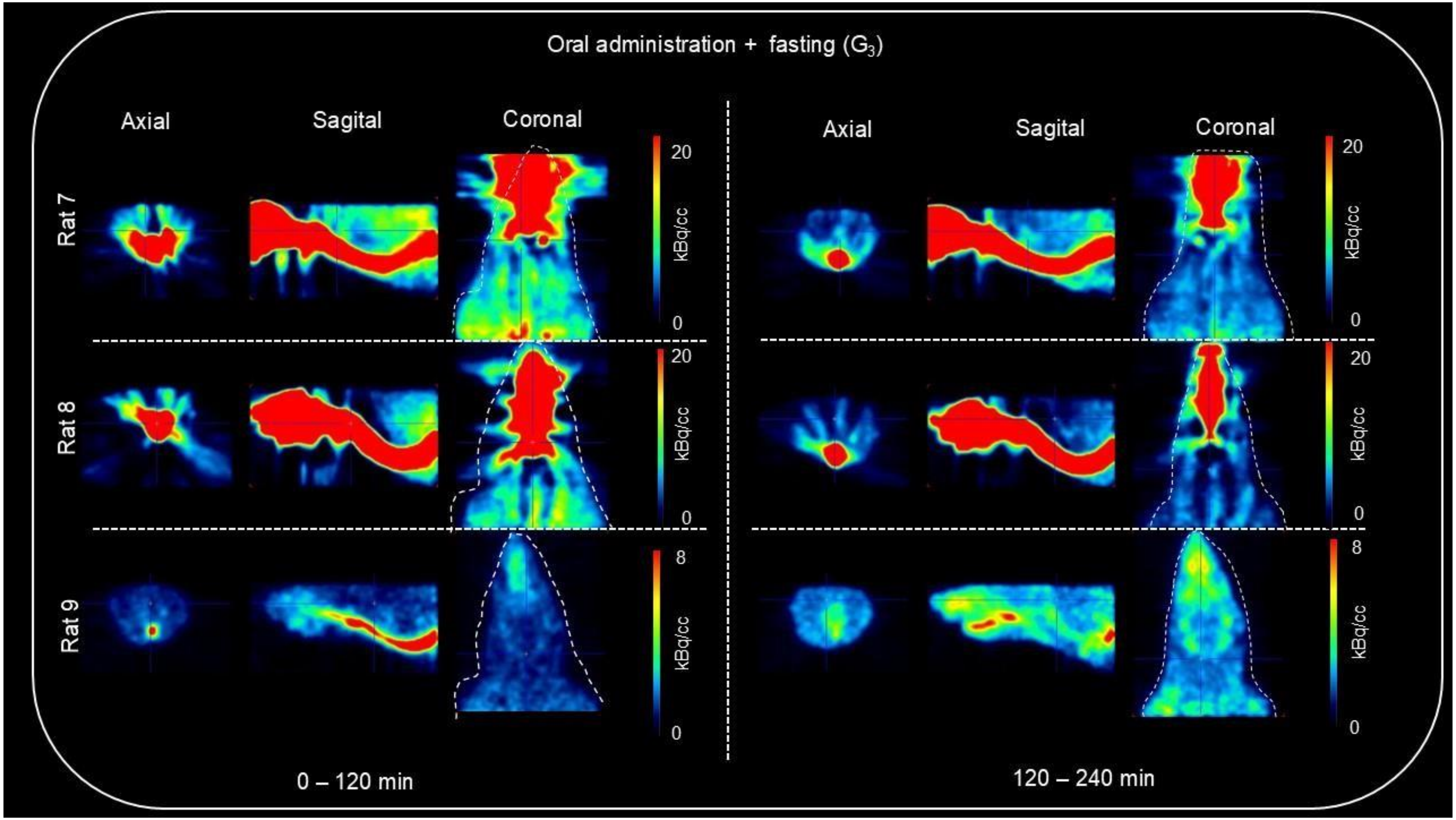

Supplementary Figure 2. Brain PET scan for group G_3. G_3: oral administration after 12 –14 h fasting.

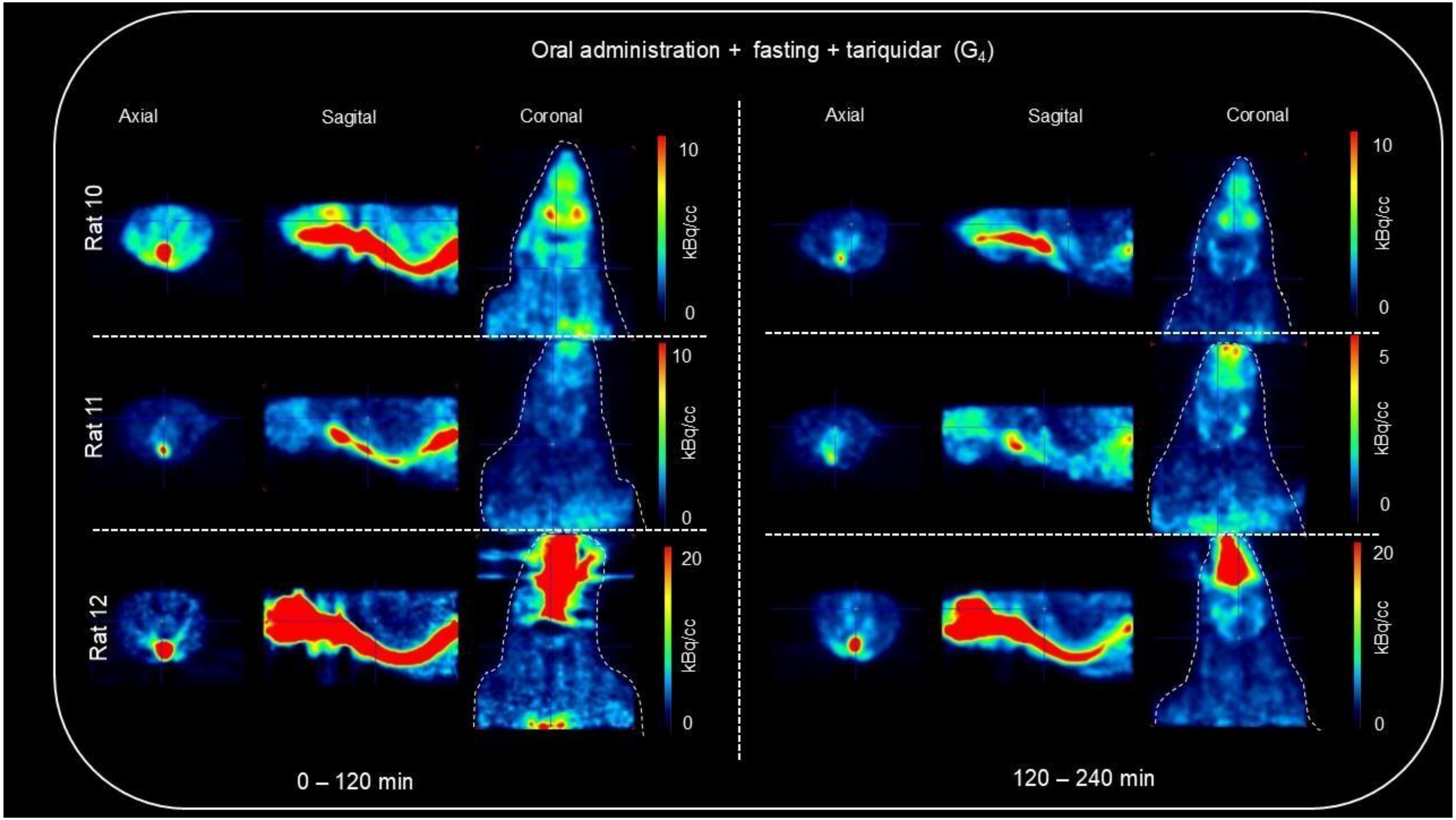

Supplementary Figure 3. Brain PET scan for group G_4. G_4: oral administration after 12–14 h fasting and tariquidar intraperitoneal injection.

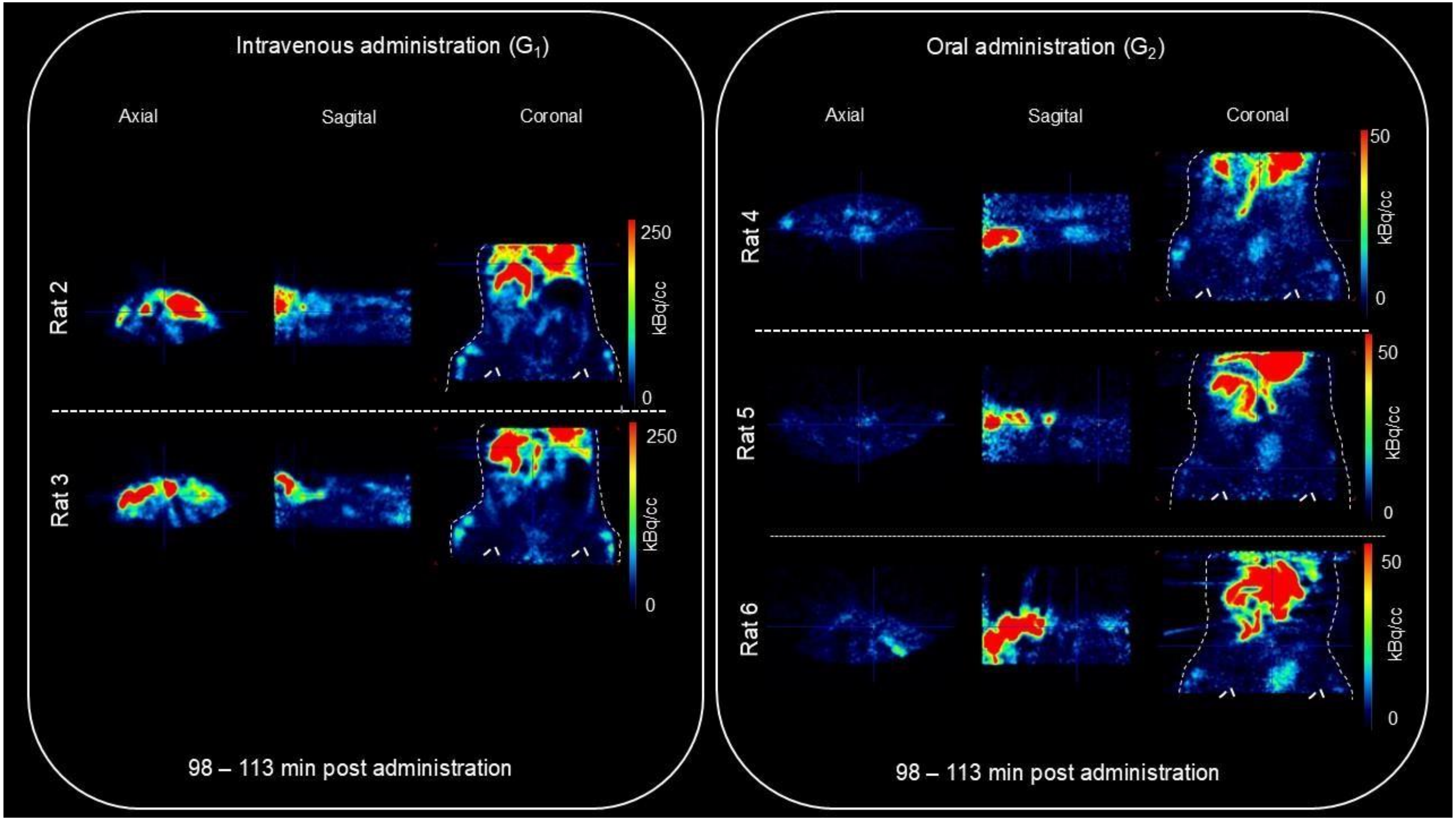

Supplementary Figure 4. Abdominal PET scan for group G_1 and G_2. G_1: intravenous injection; G_2: oral administration.

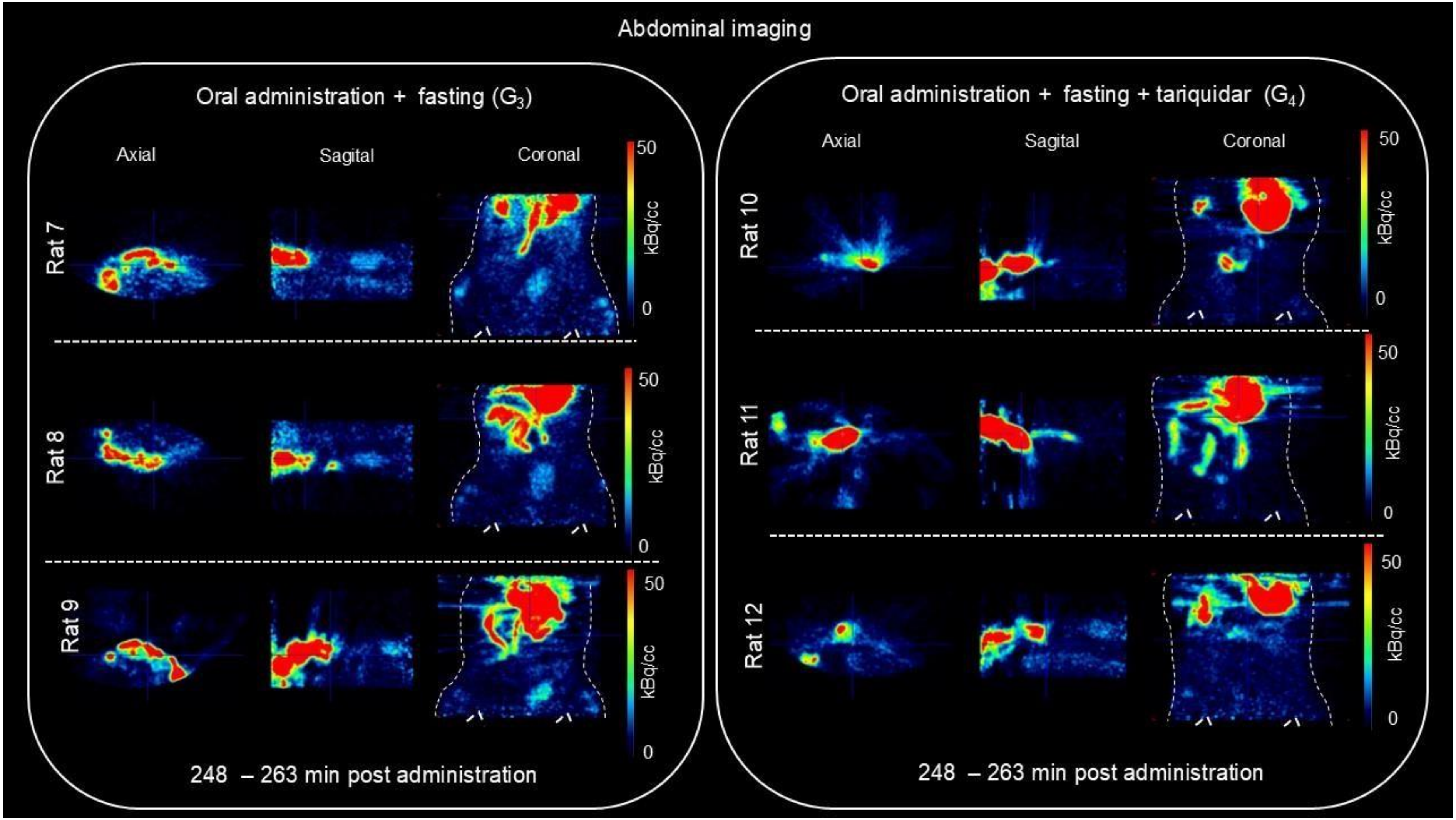

Supplementary Figure 5. Abdominal PET scan for group G_3 and G_4. G_3: oral administration after 12 –14 h fasting; G_4: oral administration after 12–14 h fasting and intraperitoneal tariquidar injection before 1 hour of tracer administration.

Supplementary Table 1. Biodistribution, in terms of kBq/g to each animal evaluated in this study

GROUP	G_1			G_2			G_3			G_4		
**REGION / ANIMAL**	**Rat 1**	**Rat 2**	**Rat 3**	**Rat 4**	**Rat 5**	**Rat 6**	**Rat 7**	**Rat 8**	**Rat 9**	**Rat 10**	**Rat 11**	**Rat 12**
Whole blood	7.2	15.1	19.2	2.3	0.3	2.5	13.1	6.4	15.1	4.9	4.4	9.9
Plasma	8.4	19.8	23.1	2.8	0.5	3.6	15.1	9.0	18.3	6.8	6.3	12.4
Heart	27.0	42.5	42.3	3.6	0.8	28.7	16.8	13.2	15.1	5.8	4.9	15.1
Lungs	80.1	130.3	155.8	92.9	34.1	50.5	75.4	78.5	24.0	9.6	8.3	51.6
Pancreas	165.7	1420.1	235.9	5.0	1.1	19.6	46.6	20.7	30.7	16.0	5.7	27.8
Spleen	130.0	204.9	210.8	9.9	1.4	10.4	32.2	34.7	20.6	6.7	5.7	15.4
Kidneys	61.7	149.7	146.2	6.9	1.4	8.9	32.1	30.7	23.4	8.9	6.9	19.8
Adrenals	189.5	287.9	321.2	15.2	3.0	13.4	49.5	50.4	22.4	15.1	5.9	30.4
Liver	206.8	337.7	372.1	11.7	1.6	61.1	67.4	19.4	125.7	12.1	19.7	42.1
Stomach	144.5	477.3	494.8	110.8	924.0	468.9	1941.8	2052.4	2150.3	1816.1	1423.7	1865.0
Duodenum	362.0	577.3	529.2	44.0	118.0	282.8	424.0	160.2	818.7	148.3	175.9	420.7
Jejunum	91.4	243.3	1834.5	7.8	3.2	121.8	46.0	63.0	618.0	158.8	138.2	701.7
Ileum	72.6	188.4	174.7	5.2	1.5	6.8	425.5	29.4	24.4	11.5	5.1	33.4
Cecum	57.6	71.5	52.6	6.1	5.5	6.8	28.6	27.7	24.8	9.7	7.1	29.5
Colon	52.6	140.0	132.3	3.3	1.6	6.2	37.3	25.7	25.6	8.9	5.4	18.9
Muscle	23.5	42.9	53.9	2.0	0.2	2.6	9.8	7.2	8.5	4.0	2.2	7.0
Femur (bone)	58.3	89.8	116.4	6.1	0.6	4.4	51.7	19.2	23.9	9.9	5.2	23.6
Midbrain	16.0	27.3	29.7	2.4	0.4	3.1	10.7	9.9	14.5	6.1	5.2	15.5
Cerebellum	16.3	24.5	27.8	2.3	0.4	2.8	12.6	9.8	13.4	6.1	4.6	14.4
Cortex	18.6	28.7	33.6	2.6	0.4	3.0	12.7	11.8	13.2	6.1	5.3	15.1

Supplementary Table 2. Tissue-to-plasma ratio to each animal evaluated in this study.

GROUP	G_1			G_2			G_3			G_4		
**REGION / ANIMAL**	**Rat 1**	**Rat 2**	**Rat 3**	**Rat 4**	**Rat 5**	**Rat 6**	**Rat 7**	**Rat 8**	**Rat 9**	**Rat 10**	**Rat 11**	**Rat 12**
Whole Blood	0.9	0.8	0.8	0.8	0.7	0.7	0.9	0.7	0.8	0.7	0.7	0.8
Heart	3.2	2.2	1.8	1.3	1.8	8.0	1.1	1.5	0.8	0.9	0.8	1.2
Lungs	9.6	6.6	6.7	32.9	74.1	14.1	5.0	8.7	1.3	1.4	1.3	4.2
Pancreas	19.9	71.8	10.2	1.8	2.3	5.5	3.1	2.3	1.7	2.4	0.9	2.2
Spleen	15.6	10.4	9.1	3.5	3.0	2.9	2.1	3.8	1.1	1.0	0.9	1.2
Kidneys	7.4	7.6	6.3	2.4	3.0	2.5	2.1	3.4	1.3	1.3	1.1	1.6
Adrenals	22.7	14.6	13.9	5.4	6.4	3.7	3.3	5.6	1.2	2.2	0.9	2.5
Liver	24.8	17.1	16.1	4.1	3.5	17.0	4.5	2.1	6.9	1.8	3.2	3.4
Stomach	17.3	24.1	21.4	39.2	2006.1	130.7	128.9	227.1	117.4	269.2	227.3	0.2
Duodenum	43.4	29.2	22.9	15.6	256.2	78.8	28.1	17.7	44.7	22.0	28.1	33.9
Jejunum	11.0	12.3	79.4	2.8	6.8	33.9	3.1	7.0	33.7	23.5	22.1	.5
Ileum	8.7	9.5	7.6	1.8	3.2	1.9	28.2	3.3	1.3	1.7	0.8	2.7
Cecum	6.9	3.6	2.3	2.2	12.0	1.9	1.9	3.1	1.4	1.4	1.1	2.4
Colon	6.3	7.1	5.7	1.2	3.5	1.7	2.5	2.8	1.4	1.3	0.9	1.5
Muscle	2.8	2.2	2.3	0.7	0.5	0.7	0.7	0.8	0.5	0.6	0.4	0.6
Femur (Bone)	7.0	4.5	5.0	2.2	1.4	1.2	3.4	2.1	1.3	1.5	0.8	1.9
Midbrain	1.9	1.4	1.3	0.9	0.8	0.9	0.7	1.1	0.8	0.9	0.8	1.2
Cerebellum	2.0	1.2	1.2	0.8	0.8	0.8	0.8	1.1	0.7	0.9	0.7	1.2
Cortex	2.2	1.5	1.5	0.9	0.8	0.8	0.8	1.3	0.7	0.9	0.9	1.2

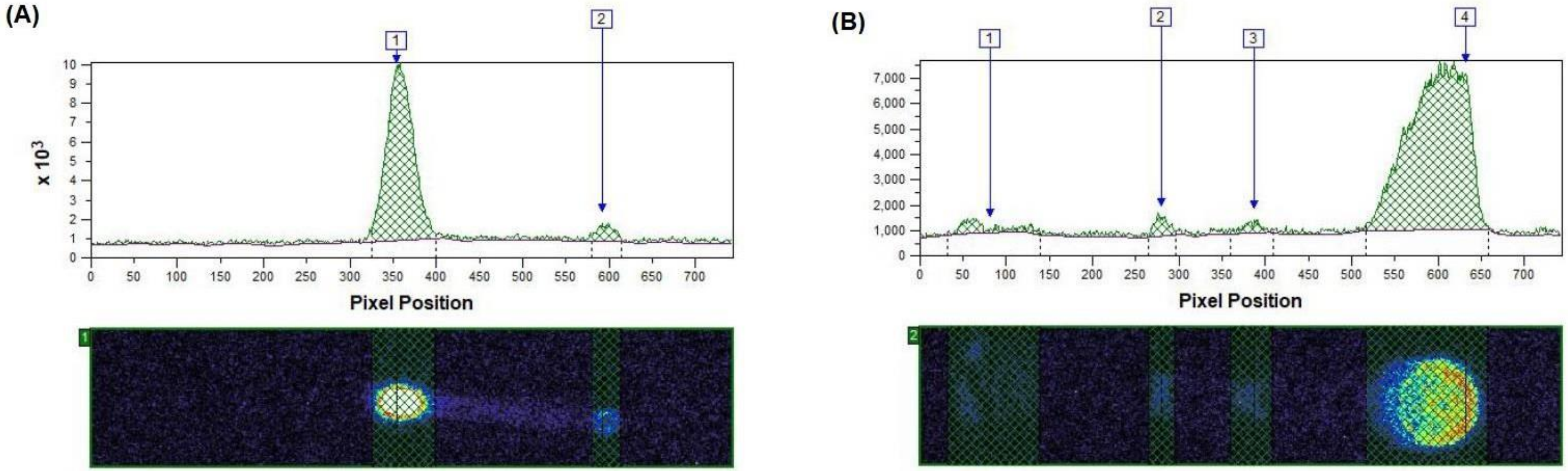

Band Table (A)

Band No	Volume	Vol+BkGnd	Band %	Lane %	Rf
1	52663410.50	63918173.00	94.06	77.59	0.477
2	3323223.00	8242948.00	5.94	4.90	0.798

Band Table (B)

Band No	Volume	Vol+BkGnd	Band %	Lane %	Rf
1	3821037.76	19311984.00	4.10	3.82	0.111
2	2429742.75	6363201.00	2.60	2.43	0.377
3	1941402.51	9052548.00	2.08	1.94	0.523
4	85091553.60	108754841.00	91.22	85.13	0.852

Supplementary Figure 6. TLC image for radiometabolites analysis. (A) standard of [^{18}F]MC225; (B) radiometabolites from plasma.
